# Immunogenicity of Oxford-AstraZeneca COVID-19 Vaccine in Vietnamese Health-Care Workers

**DOI:** 10.4269/ajtmh.21-0849

**Published:** 2022-01-07

**Authors:** Nguyen Van Vinh Chau, Lam Anh Nguyet, Nguyen Thanh Truong, Le Mau Toan, Nguyen Thanh Dung, Le Manh Hung, Mai Thanh Nhan, Dinh Nguyen Huy Man, Nghiem My Ngoc, Huynh Phuong Thao, Tran Nguyen Hoang Tu, Huynh Kim Mai, Do Thai Hung, Nguyen Thi Han Ny, Le Kim Thanh, Nguyen To Anh, Nguyen Thi Thu Hong, Le Nguyen Truc Nhu, Lam Minh Yen, Marc Choisy, Tran Tan Thanh, Guy Thwaites, Le Van Tan, for the Oxford University Clinical Research Unit COVID-19 Research Group

**Affiliations:** ^1^ Hospital for Tropical Diseases, Ho Chi Minh City, Vietnam;; ^2^ Oxford University Clinical Research Unit, Ho Chi Minh City, Vietnam;; ^3^ Institute of Pasteur, Nha Trang City, Vietnam;; ^4^ Centre for Tropical Medicine and Global Health, Nuffield Department of Medicine, University of Oxford, Oxford, United Kingdom

## Abstract

We studied the immunogenicity of the Oxford-AstraZeneca vaccine in health-care workers of a major infectious diseases hospital in Vietnam. We measured neutralizing antibodies before and 14 days after each dose, and at day 28 and month 3 after dose 1. A total of 554 workers (136 men and 418 women; age range, 22–71 years; median age, 36 years) participated with the study. Of the 144 participants selected for follow-up after dose 1, 104 and 94 gave blood for antibody measurement at weeks 6 and 8, and at month 3 after dose 1, respectively. The window time between the two doses was 6 weeks. At baseline, none had detectable neutralizing antibodies. After dose 1, the proportion of participants with detectable neutralizing antibodies increased from 27.3% (151 of 554) at day 14 to 78.0% (432 of 554) at day 28. Age correlated negatively with the development and the levels of neutralizing antibodies. However, at day 28, these differences were less profound, and women had a greater seroconversion rate and greater levels of neutralizing antibodies than men. After dose 2, these age and gender associations were not observable. In addition, the proportion of study participants with detectable neutralizing antibodies increased from 70.2% (73 of 104) before dose 2 (week 6, after dose 1) to 98.1% (102 of 104) 14 days later. At month 3, neutralizing antibodies decreased and 94.7% (89 of 94) of the study participants remained seropositive. The Oxford-AstraZeneca COVID-19 vaccine is immunogenic in Vietnamese health-care workers. These data are critical to informing the deployment of the COVID-19 vaccine in Vietnam and in Southeast Asia, where vaccination coverage remains inadequate.

## INTRODUCTION

Severe acute respiratory syndrome coronavirus 2 (SARS-CoV-2) is the cause of the ongoing COVID-19 pandemic.^1^ Since its first detection in Wuhan, China, in late 2019, SARS-CoV-2 has now become an endemic virus globally. The vaccine is thus the most plausible approach to return to pre-pandemic life. As such, vaccine development has ramped up globally during the past year. As of June 1, 2021, 185 and 102 vaccine candidates are under the pre-clinical and clinical development phases, respectively.^2^ In addition, seven vaccines have received WHO approval for emergency use.^2^

Approved vaccines have been rapidly deployed globally. As of July 26, 2021, more than 3.8 billion doses of COVID-19 vaccines have been administered worldwide. Vietnam received the first doses of the Oxford-AstraZeneca vaccine in early March 2021. As of July 18, 2021, more than 4 million doses have been administered in Vietnam, the majority of which included the Oxford-AstraZeneca vaccines.^3^

Although a vaccine must fulfill the required efficacy criteria to receive an approval for use in humans, the rapid development and deployment of COVID-19 vaccines worldwide necessitate follow-up studies to understand more fully the development and persistence of vaccine-induced immunity in different populations. Such knowledge is critical to inform global vaccination strategies and the development of next-generation vaccines.

Despite the current surge, which has been escalating since the second week of May 2021, Vietnamese people remained relatively naive to SARS-CoV-2 infections.^4^^,^^5^ As of July 18, 2021, 31,391 polymerase chain reaction-confirmed cases have been reported in Vietnam—a country of more than 97 million people.^3^ Therefore, Vietnam is an ideal setting for a vaccine evaluation study because the results naturally reflect the immunity induced by COVID-19 vaccines. There has been no report about the immunogenicity of the Oxford-AstraZeneca COVID-19 vaccine from Southeast Asia. We studied the immunogenicity of the Oxford-AstraZeneca COVID-19 vaccine in a cohort of 554 health-care workers in an infectious diseases hospital in southern Vietnam.

## METHODS

### Setting and COVID-19 vaccine rollout in Vietnam.

Our study was conducted at the Hospital for Tropical Diseases (HTD) in Ho Chi Minh City. HTD is a 550-bed tertiary referral hospital for patients with infectious diseases (including COVID-19) in southern Vietnam.^6^ Between December 2020 and February 2021, ≈40 COVID-19 patients were treated at HTD.

Vietnam received the first 117,000 doses of the Oxford-AstraZeneca COVID-19 vaccine in early March 2021. The time window between the two doses was set for a minimum of 4 weeks, with some variation (4–12 weeks), depending on the availability of the vaccine. According to the Vietnamese Ministry of Health, high-risk groups, especially frontline health-care workers, were prioritized for vaccination (Supplemental Materials). HTD staff members were eligible for vaccination and were the first in Vietnam to receive a COVID-19 vaccine in March 2021.

### Data collection.

We collected demographic information and 3 mL of blood from the study participants. Blood sampling was scheduled for a total of eight time points, including one before and seven after dose 1. The seven time points after dose 1 included weeks 2, 4, 6, and 8; and months 3, 6 and 12. Week 6 and 8 after dose 1 corresponded to the time points of before and 14 days after dose 2, respectively.

After week 4 of the first dose, because of resource constraints, blood sampling was narrowed down to a subgroup of 144 randomly selected individuals, matching for age and gender with the whole-group study participants (Table 1). Our report focuses on the period from baseline to month 3 after the first dose.

**Table 1:** Demographics of the study participants

Demographic	Whole group (*N* = 554)	Subgroup 1 (*n* = 144)	Subgroup 2 (*n* = 104)	Subgroup 3 (*n* = 94)	Comparison between groups		
					P1*	P2†	P3‡
Male, *n* (%)	136 (25)	31 (22)	25 (24)	21 (22)	0.449	0.912	0.644
Female, *n* (%)	418 (75)	113 (78)	79 (76)	73 (78)			
Median age, y (range)†	36 (22–71)	37 (24–65)	37 (24–5)	37 (24–65)	0.136	0.213	0.154
Age group, y							
20–39, *n* (%)	332 (60)	79 (55)	57 (55)	50 (53)	0.313	0.621	0.471
40–60,* n* (%)	217 (39)	64 (44)	46 (44)	43 (46)			
61–71, *n* (%)	5 (1)	1 (1)	1 (1)	1 (1)			

P*x* = *P *values per subgroup.

* Comparison between the whole group and subgroup 1.

† Comparison between whole group and subgroup 2.

‡ Comparison between whole group and subgroup 3.

### Neutralizing antibody measurement.

Neutralizing antibodies were measured using a U.S. Food and Drug Administration Emergency Use Authorization-approved assay: namely, the SARS-CoV-2 surrogate virus neutralization test (GenScript, Singapore). Prior to testing, plasma samples were first diluted 1:10 and then inactivated at 56°C for 30 minutes. The experiments were carried out according to the manufacturer’s instructions. Results are expressed as a percentage of inhibition, using a cutoff of 30%. This cutoff was applied successfully in the original report.^7^ The percentage of inhibition measured by the SARS-CoV-2 surrogate virus neutralization test has been shown to correlate well with the neutralizing antibody titers measured by the conventional plaque reduction neutralization assay.^7^

### Neutralizing antibody data from cases of natural infection.

To compare the development of neutralizing antibodies induced by vaccination against that of natural infection, we included data from 11 Vietnamese patients who had mild or asymptomatic infections. Details about these individuals and neutralizing antibody measurements are detailed in our recent report.^8^

### Statistical analysis.

We used Fisher’s exact, the χ^2^, or the Mann-Whitney *U* test to compare between groups (when appropriate). Logistic regression was used to assess the association between age and the probability of having detectable neutralizing antibodies. Linear regression was used to assess the association between age and neutralizing antibodies levels. The analyses were carried using Prism 9.0.2 (graphpad.com, San Diego, CA).

### Ethical approval.

The study was approved by the Institutional Review Board of HTD and the Oxford Tropical Research Ethics Committee, University of Oxford, UK. Written informed consent was obtained from all participants.

## RESULTS

### Demographics of the study participants.

A total of 649 of 894 HTD staff members (72.6%) consented to participate in the vaccine evaluation study. Five hundred fifty-four of 649 participants (85.4%) were monitored successfully up to day 28 after the first dose and were thus included for analysis as a whole group. The 554 study participants were between 22 and 71 years of age (median age, 36 years). Women were predominant, accounting for 75.4% of study participants (418 of 554) (Table 1).

Of the 144 participants in the subgroup, 104 (72.2%) and 94 (65.3%) were monitored successfully up to 14 days after the second dose (i.e., week 8 after dose 1) and 3 months (week 13) after the first dose, respectively. The age and gender distributions of these subgroups are comparable with that of the whole group (Table 1). The time window between the first and the second dose was 6 weeks.

### Development of detectable neutralizing antibodies.

At baseline, none of the 104 study participants in the subgroup had detectable neutralizing antibodies (Table 2). At days 14 and 28 after the first dose, the proportion of study participants with detectable neutralizing antibodies increased from 27.3% (151 of 554) to 78.0% (432 of 554), respectively, among all 554 individuals of the whole group. The proportion of study participants with detectable neutralizing antibodies reached 98.1% (102 of 104) 14 days after the second dose, and then decreased slightly to 94.7% (89 of 94) at month 3 after the first dose (Table 2).

**Table 2:** The proportion of study participants with detectable neutralizing antibodies after vaccination

Time point	Whole group				Subgroup			
	Total (*N* = 554)	Male (*n* = 136)	Female (*n* = 418)	*P* value*	Total (*N* = 104)	Male (*n* = 25)	Female (*n* = 79)	*P* value*
Baseline, *n* (%)	0	0	0	NA	0	0	0	NA
14 Days after dose 1, *n* (%)	151 (27.3)	40 (29.4)	111 (26.6)	0.52	31 (29.8)	10 (40.0)	21 (26.6)	0.20
28 Days after dose 1, *n* (%)	432 (78.0)	97 (71.3)	335 (80.1)	0.031	82 (78.8)	20 (80.0)	62 (78.5)	0.87
Before dose 2, *n* (%)	NA	NA	NA	NA	73 (70.2)	17 (68.0)	56 (70.1)	0.78
14 Days after dose 2*, n* (%)	NA	NA	NA	NA	102 (98.1)	24 (96.0)	78 (98.7)	0.43
Month 3 after the first dose†	NA	NA	NA	NA	89 (94.7)	21 (95.5)	68 (94.4)	1

NA = not applicable.

* For comparison between men and women.

† *N* = 94 (22 men and 72 women).

### Kinetics of neutralizing antibody levels.

**Figure 1.**
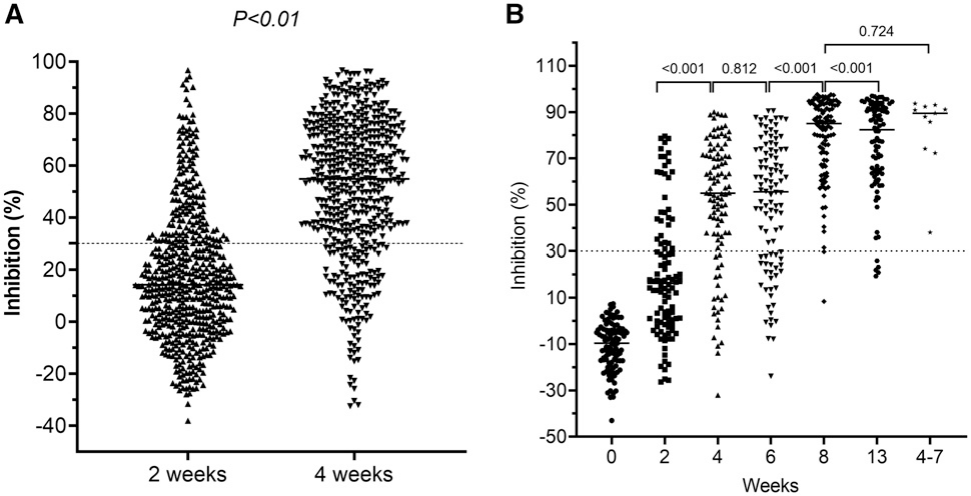
Development of neutralizing antibodies levels after vaccination. (**A**) Neutralizing antibody levels measured at 2 and 4 weeks after the first dose was administered to 554 study participants. (**B**) Neutralizing antibody levels measured at time points from baseline to month 3 after the first dose was administered to the subgroups. Data on neutralizing antibody levels obtained from 11 convalescent sera collected during weeks 4 through 7 (right column) from cases with mild or asymptomatic infection were included as references.

After the first dose, neutralizing antibody levels measured at day 28 were significantly greater than those measured on day 14 (Figure 1A), but comparable with those measured at week 6 (Figure 1B). On day 14 after the second dose, neutralizing antibodies increased significantly and were comparable with those levels obtained from Vietnamese people with asymptomatic or mild infection (Figure 1B). At month 3 after the first dose, neutralizing antibody levels were significantly less than those measured at 14 days after the second dose (Figure 1B).

### Neutralizing antibodies versus age and gender.

**Figure 2.**
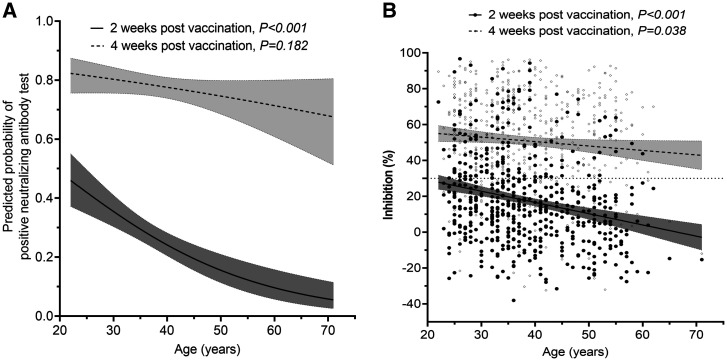
The associations between neutralizing antibody level and age. (**A**) Association between age and the probabilities of having detectable neutralizing antibodies at 2 and 4 weeks after the first does was administered to 554 study participants. (**B**) Association between age and neutralizing antibody levels measured at 2 and 4 weeks after the first dose was administered to 554 study participants. Black circles represent data for the 14-day time point; gray circles represent data for 28-day time point. Shaded areas indicate 95% CIs.

**Figure 3.**
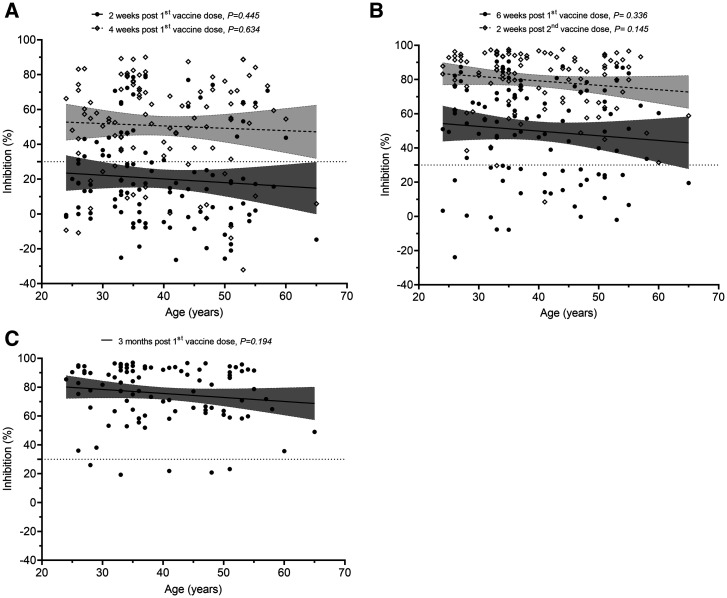
Neutralizing antibody levels of participants selected to assess the impact of the second dose. (**A**) Two and 4 weeks after the first dose (*n* = 104). (**B**) Before the second dose (6 weeks after the first dose) and 2 weeks after the second dose (*n* = 104). (**C**) At month 3 after the first dose (*n* = 94). Shaded areas indicate 95% CIs.

On day 14 after the first dose, the development and levels of detectable neutralizing antibodies among the 554 study participants correlated negatively with age. This difference was less profound at day 28 after dose 1, especially with regard to the development of detectable neutralizing antibodies (Figure 2). At these corresponding time points, similar trends were also observed among individuals of the subgroup, but the difference was not significant (Figure 3 and Supplemental Figure 1), likely because of the small sample size. At 14 days after the second dose (week 8 after dose 1) and month 3 after the first dose, the proportion of individuals with detectable neutralizing antibodies was similar across age groups (Supplementa1 Figure 1B and C).

In terms of gender, with the exception of day 28 after the first dose, neutralizing antibody levels and the proportion of study participants with detectable neutralizing antibodies were comparable between men and women (Table 2 and Supplemental Figure 2).

## DISCUSSION

We report the immunogenicity of the Oxford-AstraZeneca COVID-19 vaccine in a cohort of 554 Vietnamese health-care workers in a tertiary referral hospital for patients with infectious diseases in southern Vietnam. We show that the Oxford-AstraZeneca COVID-19 vaccine is immunogenic in Vietnamese people. Neutralizing antibodies increased after each dose, with the seroconversion rate reaching 98.1% (102 of 104) at 14 days after dose 2. At month 3 after dose 1, neutralizing antibody levels decreased, and 94.7% (89 of 94) of the study participants remained seropositive.

Findings from the original phase 2/3 trial showed that spike protein-specific IgG developed within 2 weeks after vaccination; and at 14 days after the second dose, its titers increased, with a seroconversion rate of 208 of 209 (> 99%).^9^ Consistently, our study shows the development and levels of neutralizing antibodies increase significantly after each dose, with the former reaching 98.1% at 14 days after the second dose. Parallel with these reports are real-word data from the United Kingdom showing that the administration of the second dose increased protection against SARS-CoV-2 infection from 65% by dose 1 to 70% by dose 2 among vaccine recipients.^10^ A single dose of Oxford-AstraZeneca or Pfizer COVID-19 vaccines reduced COVID-19 hospital admissions among vaccine recipients by 88% and 91%, respectively in Scotland.^11^

Older individuals, especially those 80 years or older, without prior infection had lower levels of neutralizing antibodies induced by the first dose than younger adults.^12^^,^^13^ These age-dependent responses were most profound within the first 3 weeks after vaccination, but were resolved by the administration of the second dose.^12^ Although similar trends were observed in our study, at day 28 after the first dose, the differences in our study were negligible, especially in terms of the seroconversion rate. None of our study participants was older than 71 years, which explains why the observed differences were less profound compared with the UK population-based study.^10^

Our results provide reassuring evidence for the effectiveness of the proposed vaccination strategy that aims at prioritizing the first dose for as many people as possible in the first instance.^14^ However, the data also emphasize the importance of the second dose,^15^ especially in older people, and it remains unknown whether the third dose is needed to provide long-term protection. A decline in antibody titers was recorded at weeks 8 through 12 after the first two doses among 75 study participants in the United Kingdom,^16^ but the administration of a third dose helped boost the immune response. Antibody waning is presumably more profound among individuals without prior infection. A follow-up study is therefore critical to assess the levels of antibody waning among our study participants and the correlated level of protection, especially in the context of the rapid spread of the delta variant globally.

A strength of our study is that it was conducted in a naive population, with no prior SARS-CoV-2 infections.^4^ Thus, our data reflect more naturally the immunity profiles induced by the Oxford-AstraZeneca COVID-19 vaccine. In addition, although the correlates of protection for the COVID-19 vaccine remain to be determined, neutralizing antibodies are considered to be the most reliable surrogates.^17^ Therefore, by measuring neutralizing antibodies, our findings reflect more accurately the potential of correlates of protection.

Our study has some limitations. First, we did not study cellular immunity, especially T-cell response. Cellular immunity has been recognized increasingly to play a role in the pathogenicity and immune response of the SARS-CoV-2 infection.^18^ Therefore, the durability of both cellular and humoral immune responses should be explored further. Second, because of the age and gender structure of the HTD staff, we did not include participants older than 71 years, and women were predominant among our study subjects. Of note, compared with men, women seemed to respond better to the Oxford-AstraZeneca COVID-19 vaccine at day 28 after the first dose, which merits further research.

In summary, the Oxford-AstraZeneca COVID-19 vaccine is immunogenic in Vietnamese health-care workers. Neutralizing antibody levels decreased at month 3 after vaccination. The requirement for a third dose warrants further research. These data are critical to informing the deployment of the COVID-19 vaccine in Vietnam and beyond.

## Supplemental Material


Supplemental materials
