# Supplementary material for: Immunogenicity of Oxford-AstraZeneca COVID-19 Vaccine in Vietnamese Health-Care Workers
Source: Am J Trop Med Hyg. 2022 Jan 7;106(2):556–61. doi: 10.4269/ajtmh.21-0849 (PMC8832887; doi:10.4269/ajtmh.21-0849)
Supplement: Supplementary file 1 [file tpmd210849.SD1.pdf]

Immunogenicity of Oxford-AstraZeneca COVID-19 vaccine in Vietnamese healthcare workers

Nguyen Van Vinh Chau[1], Lam Anh Nguyet[2], Nguyen Thanh Truong[1], Le Mau Toan[1], Nguyen Thanh Dung[1], Le Manh Hung[1], Mai Thanh Nhan[1], Dinh Nguyen Huy Man[1], Nghiem My Ngoc[1], Huynh Phuong Thao[1], Tran Nguyen Hoang Tu[1], Huynh Kim Mai[3], Do Thai Hung[3], Nguyen Thi Han Ny[2], Le Kim Thanh[2], Nguyen To Anh[2], Nguyen Thi Thu Hong[2], Le Nguyen Truc Nhu[2], Lam Minh Yen[2], Marc Choisy[2,4], Tran Tan Thanh[2], Guy Thwaites[2,3] and Le Van Tan[2], for OUCRU COVID-19 Research Group*

[1]Hospital for Tropical Diseases, Ho Chi Minh City, Vietnam
[2]Oxford University Clinical Research Unit, Ho Chi Minh City, Vietnam
[3]Institute of Pasteur, Nha Trang City, Vietnam
[4]Centre for Tropical Medicine and Global Health, Nuffield Department of Medicine, University of Oxford, Oxford, UK

*Members of the groups are listed in the acknowledgments.

Correspondence: Nguyen Van Vinh Chau, chaunvv@oucru.org, Le Van Tan, tanlv@oucru.org

List of groups prioritized for COVID-19 vaccination in Vietnam

1. Frontline healthcare workers of COVID-19, including people whose work is to deal with COVID-19 prevention and control work (members of COVID-19 steering committees at all levels, staff at state-run quarantine sites, people conducting contract tracing and epidemiological investigations, volunteers, reporters among others), military and public security forces
2. Vietnamese diplomats, customs and immigration officers
3. Essential service workers in sectors such as aviation, transport, tourism, electricity and water supply
4. Teachers and individuals working at education and training facilities, and those working at State agencies with regular contact with various people
5. People with chronic diseases or aging above 65
6. Residents in outbreak hotspots in Vietnam
7. Poor people, Policy beneficiaries
8. Those who will be sent abroad for learning and working
9. Other people determined by the Ministry of Health

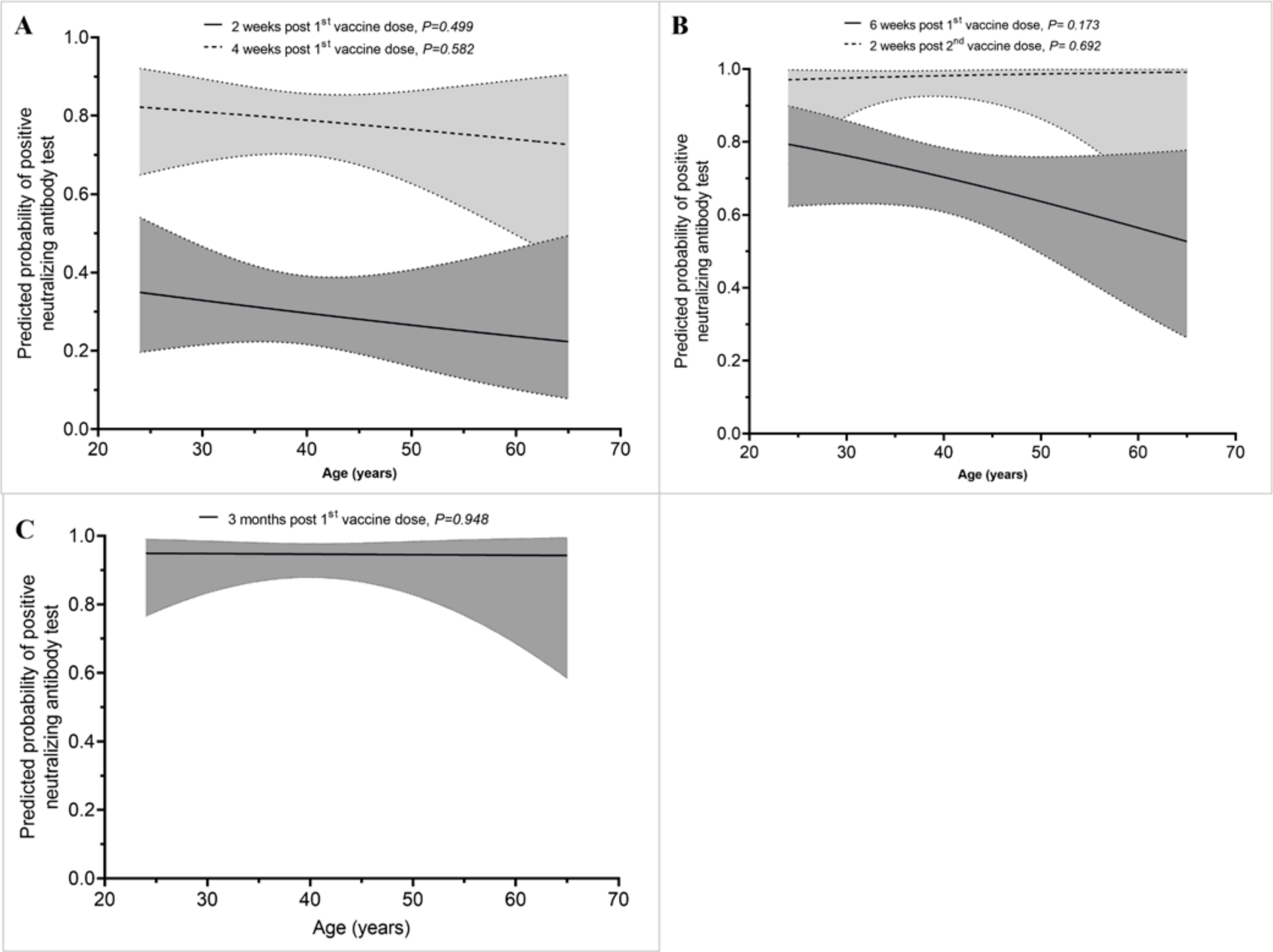

Supplementary Figure 1: Probability of having detectable neutralizing antibodies among the study participants selected for assessment of the impact of the second dose. **A**) At 2 and 4 weeks after the first dose (n=104), **B**) Before the second dose (i.e.6 weeks after the first dose) and 2 weeks after the second dose (n=104) and **C**) at month 3 after the first dose (n=94). Shaded areas indicate 95% of confident intervals.

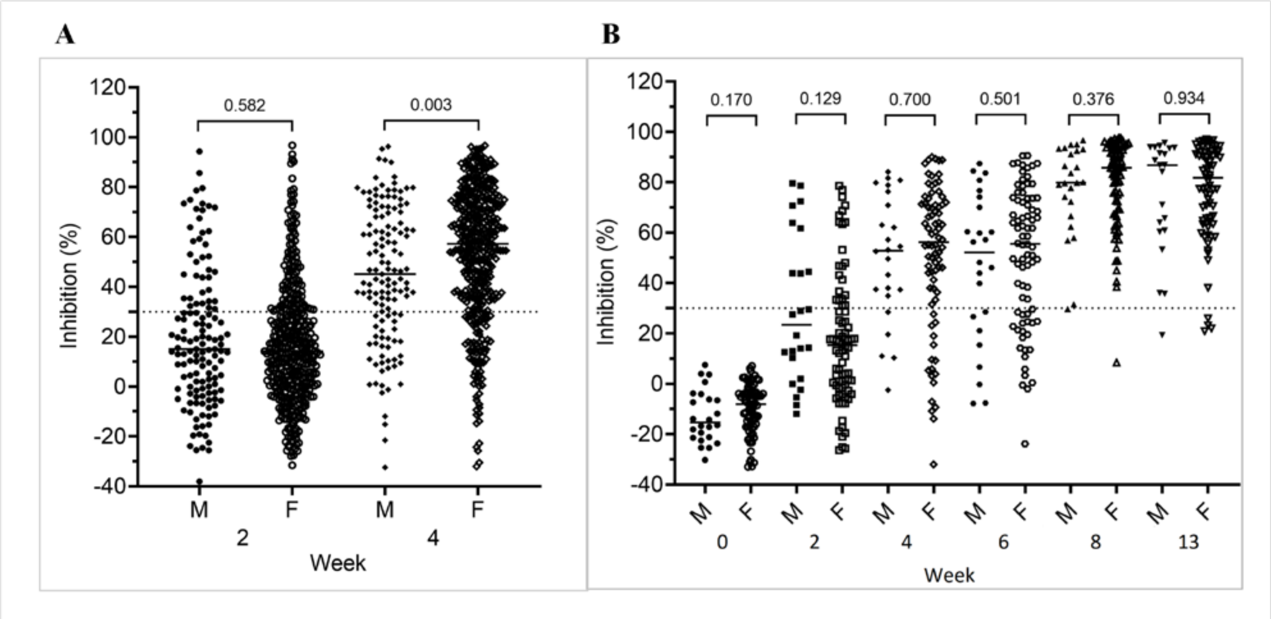

Supplementary Figure 2: Association between neutralizing antibody levels and gender. **A**) At 2 and 4 weeks after the first dose of the whole group (n=554), **B**) From baseline to month three after the first dose of the subgroup
